# Predictors of Mortality in Patients With Transcatheter Aortic Valve Implantation: A National Inpatient Sample Database Analysis

**DOI:** 10.7759/cureus.14344

**Published:** 2021-04-07

**Authors:** Arslan Inayat, Sakina Abbas, FNU Salman

**Affiliations:** 1 Internal Medicine, University at Buffalo, Catholic Health System, Buffalo, USA; 2 Medicine, Civil Hospital Karachi, Dow University of Health Sciences, Karachi, PAK; 3 Medicine, Mercy Health St. Vincent Medical Center, Toledo, USA

**Keywords:** tavi, tavr, aortic stenosis, transcatheter aortic valve implantation, transcatheter aortic valve replacement

## Abstract

Introduction

In recent decades, transcatheter aortic valve implantation (TAVI) has become a treatment of choice for aortic stenosis. The purpose of this study was to evaluate predictors of mortality in patients undergoing TAVI.

Methods

The National Inpatient Sample database from the year 2011 to 2018 was used to identify all patients undergoing TAVI during the study period.

Results

A total of 215,983 weighted hospitalizations for TAVI were included in the analysis. We report the following three main findings from our contemporary analysis of the NIS: (1) despite TAVI patients having a high comorbidity burden, mortality remains low at 2.2%, (2) in terms of baseline characteristics, end-stage renal disease, liver disease, congestive heart failure, chronic obstructive pulmonary disease, atrial fibrillation, and lung cancer remain significant predictors of mortality in patients undergoing TAVI, and (3) length of stay and cost of stay are significantly higher in patients who died during the hospitalization.

Conclusion

In conclusion, we report that at baseline, end-stage renal disease, liver disease, atrial fibrillation, and lung cancer are significant predictors of mortality in patients undergoing TAVI.

## Introduction

In the recent decade, transcatheter aortic valve implantation (TAVI) has emerged as a treatment of choice for aortic valve intervention in prohibitive and low surgical risk candidates [1-3]. TAVI has led to constant improvement in clinical outcomes with improved techniques in patients with aortic stenosis [4]. Consistent improvements in device selection, and procedural and technical performance have been made, but still TAVI is associated with risks and complications that have a strong impact on both early and long-term outcomes [5]. Most of the patients for TAVI have a high comorbidity burden and are at high risk of death and adverse events. In order to improve patient selection and to give patients a better basis for informed decision-making, identifying predictors of early mortality is important [6]. TAVI has shown to reduce mortality and improve both functional status and quality of life in older patients with aortic stenosis [7]. Logistic EuroSCORE (European System for Cardiac Operative Risk Evaluation) and the Society of Thoracic Surgeons Predicted Risk of Mortality (SRS-PROM) algorithm are the two most frequently used death-prediction models [8]. The purpose of this study was to evaluate predictors of mortality in patients undergoing TAVI from a large inpatient national database.

## Materials and methods

The National Inpatient Sample (NIS) is a publicly available inpatient billing database made to provide national estimates of hospital discharges in the United States  [3]. The NIS data from the years 2011 to 2018 were used to extract TAVI cases performed during the study time period. International Classification of Diseases, Ninth Revision (ICD-9) (3505, 3506) and ICD, Tenth Revision (ICD-10) (02RF3) codes were utilized to identify all cases of TAVI. Patients under the age of 18 years were excluded from the analysis. Baseline characteristics were compared for patients who died versus those who survived the hospitalization. The chi-square test was used for categorical variables and the Mann-Whitney U test was used for all continuous variables. A binary multivariable logistic regression was performed using baseline comorbidities including coronary artery disease (CAD), congestive heart failure (CHF), chronic obstructive pulmonary disease (COPD), cerebrovascular disease (CVD), end-stage renal disease (ESRD), obesity, peripheral vascular disease (PVD), weight loss, anemia, thrombocytopenia, breast cancer, lung cancer, alcohol use, coagulopathy, and atrial fibrillation (AF) to compute an adjusted odds ratio for predicting mortality in TAVI. Given the de-identified and public nature of the database, Institutional Review Board approval was not required for this project. As per the HCUP (Healthcare Cost and Utilization Project) reporting guidelines, observations of less than 11 cases were excluded from the analysis. All statistical analyses were performed using SPSS Version 27 (IBM Corp., Armonk, NY, USA).

## Results

**Figure 1:**
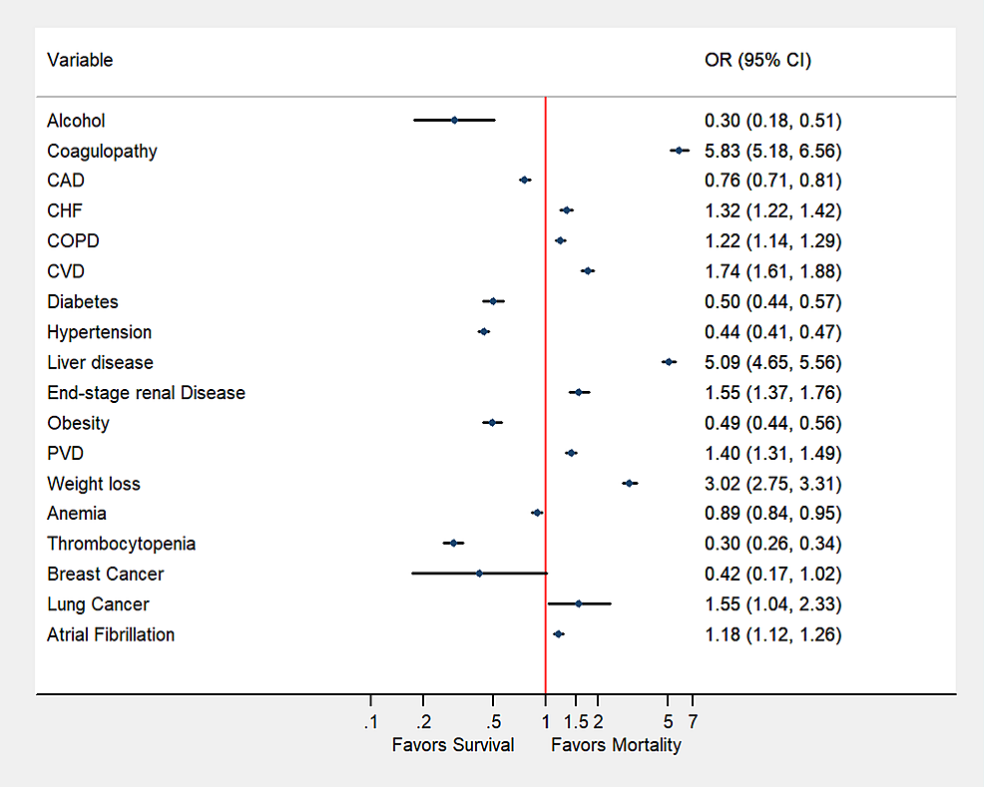
Adjusted odds of predictors of in-hospital mortality in patients undergoing transcatheter aortic valve intervention CAD, coronary artery disease; CHF, congestive heart failure; COPD, chronic obstructive pulmonary disease; CVD, cerebrovascular disease; PVD, peripheral vascular disease

A total of 215,983 weighted hospitalizations for TAVI were included in the analysis. Of the patient undergoing the procedure, 4,664 (2.2%) died and 211,319 (97.8%) survived the procedure. The detailed baseline characteristics are summarized in Table 1. At baseline, patients with CHF (OR: 1.32; 95% CI: 1.22-1.42), COPD (OR: 1.22; 95% CI: 1.14-1.29), liver disease (OR: 5.09; 95% CI: 4.65-5.57), ESRD (OR: 1.55; 95% CI: 1.37-1.76), PVD (OR: 1.40; 95% CI: 1.31-1.49), lung cancer (OR: 1.56; 95% CI: 1.04-2.33), and AF (OR: 1.18; 95% CI: 1.12-1.26) had higher adjusted rates of odds of mortality (Table 1, Figure 1). Patients who died during hospitalization had a higher median cost of stay (US$30,9412 vs. US$18,0644) and length of stay (7 vs. 3 days).

**Table 1:** Baseline characteristics of patients who survived compared to those who died undergoing transcatheter aortic valve intervention CAD, coronary artery disease; CHF, congestive heart failure; COPD, chronic obstructive pulmonary disease; CVD, cerebrovascular disease; PVD, peripheral vascular disease

Univariate analysis	Died during hospitalization		Multivariate analysis, OR (95% CI)
Variable (%)	No (211,319)	Yes (4,664)	Survived versus died
Age, median (IQR)	82 (75-87)	83 (77-88)	-
Female	98,131 (46.4%)	2,514 (53.9%)	0.74 (0.65-0.84) (reference to males)
White	175,097 (87.0%)	3,815 (86.6%)	Reference to whites
Black	8,468 (4.2%)	125 (2.8%)	0.8 (0.56-1.17)
Hispanics	9,362 (4.6%)	265 (6.0%)	0.54 (0.32-0.93)
Alcohol	722 (0.3%)	15 (0.3%)	0.3 (0.18-0.51)
Coagulopathy	28,616 (13.5%)	1,414 (30.3%)	5.827 (5.18-6.56)
CAD	145,538 (68.9%)	2,794 (59.9%)	0.76 (0.71-0.81)
CHF	155,292 (73.5%)	3,694 (79.2%)	1.315 (1.22-1.42)
COPD	70,788 (33.5%)	1,843 (39.5%)	1.216 (1.14-1.29)
CVD	24,603 (11.6%)	866 (18.6%)	1.74 (1.61-1.88)
Diabetes	26,880 (12.7%)	250 (5.4%)	0.5 (0.44-0.57)
Hypertension	184,336 (87.2%)	3,360 (72.0%)	0.44 (0.41-0.47)
Liver disease	6,280 (3.0%)	750 (16.1%)	5.09 (4.65-5.57)
End-stage renal Disease	7,893 (3.7%)	310 (6.6%)	1.55 (1.37-1.76)
Obesity	28,920 (13.7%)	285 (6.1%)	0.49 (0.44-0.56)
PVD	47,494 (22.5%)	1,375 (29.5%)	1.39 (1.31-1.49)
Weight loss	7,217 (3.4%)	665 (14.3%)	3.02 (2.75-3.31)
Anemia	63,871 (30.2%)	1,564 (33.5%)	0.89 (0.84-0.95)
Thrombocytopenia	29,686 (14.0%)	1,065 (22.8%)	0.29 (0.26-0.34)
Colorectal cancer	340 (0.2%)	0 (0.0%)	-
Breast cancer	635 (0.3%)	5 (0.1%)	0.42 (0.17-1.02)
Lung cancer	780 (0.4%)	25 (0.5%)	1.56 (1.04-2.33)
Atrial fibrillation	87,361 (41.3%)	2,201 (47.2%)	1.18 (1.12-1.26)
Income			
0-25th percentile	44,256 (21.3%)	984 (21.6%)	Reference to 0-25th percentile
25th–50th	52,967 (25.5%)	1,075 (23.6%)	0.96 (0.79-1.15)
50th-75th	55,224 (26.5%)	1,210 (26.5%)	0.87 (0.73-1.05)
75th-100th	55,648 (26.7%)	1,295 (28.4%)	0.94 (0.79-1.13)
Length of stay, median (IQR)	3 (2-6)	7 (2-16)	-
Cost of stay, median (IQR)	180,644 (133,645-257,922.8)	309,412 (208,125-455,263)	-

## Discussion

The most common valvular heart disease in the developed countries is aortic stenosis, and about 7% of the population that is over the age of 65 years suffer from this disease [8]. A life-saving, increasingly attractive, and minimally invasive treatment in severe aortic valve stenosis patients is TAVI. In the last decade, the evolution of TAVI has proven to be successful in improving the quality of life [9]. Two recent trials - the Placement of AoRTic TraNscathetER (PARTNER) Valve Trial (Edwards SAPIEN Transcatheter Heart Valve) and the CoreValve US Pivotal - have established that the TAVR procedure is the preferred approach for patients who are at high surgical risk [8]. TAVI has not only expanded to high-risk patients but also to younger and healthier patients who are at lower surgical risk [10]. Despite the improvement in survival conferred by the procedure, the main concern around the complications of this procedure is the major bleeding events that have been associated with poorer outcomes, including higher mortality within the following months of the procedure [11,12]. As compared to patients with intermediate surgical risk, patients who were at low surgical risk had a reduced risk of disabled stroke, all-cause mortality, and a composite of disabled stroke at 12 months or all-cause mortality because of TAVI [13].

We report the following three main findings from our contemporary analysis of the NIS: (1) despite TAVI patients having a high comorbidity burden, mortality remains low at 2.2%, (2) in terms of baseline characteristics, ESRD, liver disease, CHF, COPD, AF, and lung cancer remain significant predictors of mortality in patients undergoing TAVI, and (3) length of stay and cost of stay are significantly higher in patients who died during the hospitalization.

ESRD, which was not represented in the landmark PARTNER trial [1], is associated with higher odds of mortality and is well described in previous literature [14,15]. We also report that the presence of AF is an independent predictor of mortality. Previously, Zweiker et al. reported that the presence of AF as a comorbidity is associated with higher one-year mortality when compared to surgical aortic valve intervention [16]. In terms of malignancies, lung cancer is one of the most frequent cancers seen in the U.S. population and we report that it is associated with the significantly elevated mortality rate in TAVI patients. This is contrary to a previous study that reported that active cancer patients might have similar outcomes to non-cancer patients undergoing TAVI [17].

The findings of our study are not without any limitations. Given the retrospective nature of the study, the association should not be interpreted as causality. We also did not have laboratory data, or medication data, or risk scores such as STS (Society of Thoracic Surgeons). We could only evaluate in-hospital outcomes, and long-term follow-up data are not available. Coding errors can also not be excluded as our analysis is based on an administrative billing database. Interestingly, our study reported lower mortality with baseline comorbidities such as diabetes, CAD, and hypertension. However, the results of our updated NIS analysis are in agreement with previously reported literature on predictors of mortality in TAVI [10].

## Conclusions

In conclusion, we report that at baseline end-stage renal disease, liver disease, AF, and lung cancer are significant predictors of mortality in patients undergoing TAVI. As TAVI expands to a rapidly aging population, it is of utmost importance to identify patients at a high risk of developing complications.
